# Unusual primary osseous Hodgkin’s lymphoma: A case report

**DOI:** 10.3892/ol.2014.2724

**Published:** 2014-11-20

**Authors:** WEI LUO, FANGJIE ZHANG, JINPENG SUN, HONGBO HE

**Affiliations:** Department of Orthopedics, Xiangya Hospital, Central South University, Changsha, Hunan 410008, P.R. China

**Keywords:** Hodgkin’s lymphoma, bone, diagnosis

## Abstract

Hodgkin’s lymphoma (HL) is one of the few adult malignancies that most frequently presents with a progressive, painless enlargement of the peripheral lymph nodes. A primary osseous presentation of HL, without lymph node involvement, is extremely rare. The present study describes a case of primary multifocal osseous HL in a 22-year-old female. The patient presented with pain in the lumbar-sacral-pelvic area and a prolonged fever. Pathological examination led to a diagnosis of primary multifocal osseous lymphoma, and the patient was subsequently prescribed a course of Adriamycin, bleomycin, vinblastine and dacarbazine (ABVD) chemotherapy. Following this, the patient recovered with no pain or fever, and computed tomography identified no further progression. The clinical, radiological and histological features of HL are similar to those of other medical conditions, such as tuberculosis and eosinophilic granuloma. Furthermore, in rare cases, HL may even occur in combination with multiple myeloma. This makes it difficult to diagnose the condition, which often leads to a delay in treatment. Clinicians should not ignore HL when it manifests in the unusual primary osseous form.

## Introduction

Hodgkin’s lymphoma (HL) is one of the few adult malignancies that is usually curable (1). HL frequently presents as a progressive, painless enlargement of the peripheral lymph nodes, particularly around the cervical region (2). Classical HL is defined as a well-established, proliferative neoplasm of the lymph nodes that is composed of mononuclear Hodgkin cells and multi-nucleated Reed-Sternberg (RS) cells in variable proportions, along with neutrophils, eosinophils, histiocytes, fibroblasts, collagen fibers, non-neoplastic lymphocytes and plasma cells (3,4). Extra-nodal forms of HL are rare, accounting for <1% of all HL cases (5,6). HL is a systemic disease, with 10–20% of HL patients demonstrating bone involvement throughout disease progression (7–9). However, patients presenting with primary HL of the bone are unusual (2,10). The histological, radiological and clinical features of HL are similar to those of other medical conditions, including tuberculosis and eosinophilic granuloma, and in unusual cases, may even present in combination with multiple myeloma (1,2,7,9,10). This makes HL difficult to diagnose and often leads to delays in treatment.

The present study describes the case of a 22-year-old female diagnosed with primary multifocal osseous HL. Informed consent was provided by the patient and the patient’s family.

## Case report

A 22-year-old female patient presented to the Xiangya Hospital (Central South University, Changsha, Hunan, China) with a five-month history of pain in the lumbar-sacral-pelvic area, which gradually involved the left hip and subsequently involved the left shoulder. The patient’s symptoms were accompanied by a prolonged fever (a recurrent fever type, with a temperature of >39°C for 1–2 days prior to returning to normal and recurring repeatedly over the 5-month period), night sweats and weight loss. The physical examination was mostly normal, with normal appearance and range of motion. However, pain and tenderness were evident upon percussion of the lumbosacral area, left hip joint and left shoulder. The patient was previously healthy, with no exposure to contaminated water or poisons, and no communicable diseases. The patient’s parents were also healthy, with no history of a hereditary or similar disease in their families.

**Figure 1:**
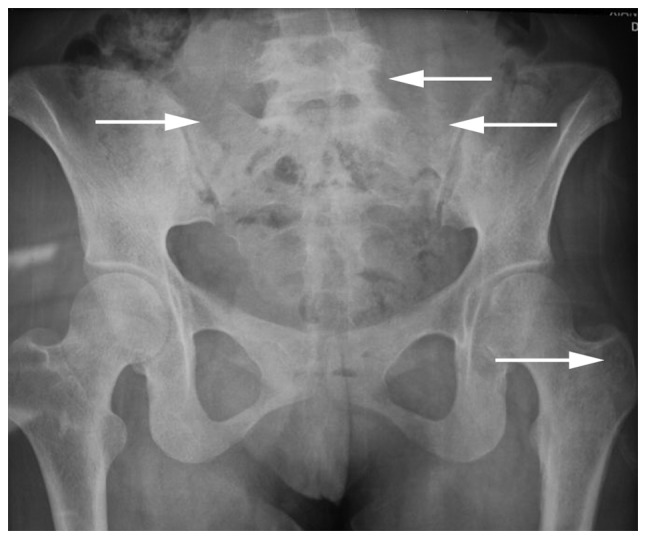
Pelvic X-ray film revealing a bone lesion (white arrows) in each sacroiliac joint and in the inferior segment of the lumbar spine. The left greater trochanter is not visible.

**Figure 2:**
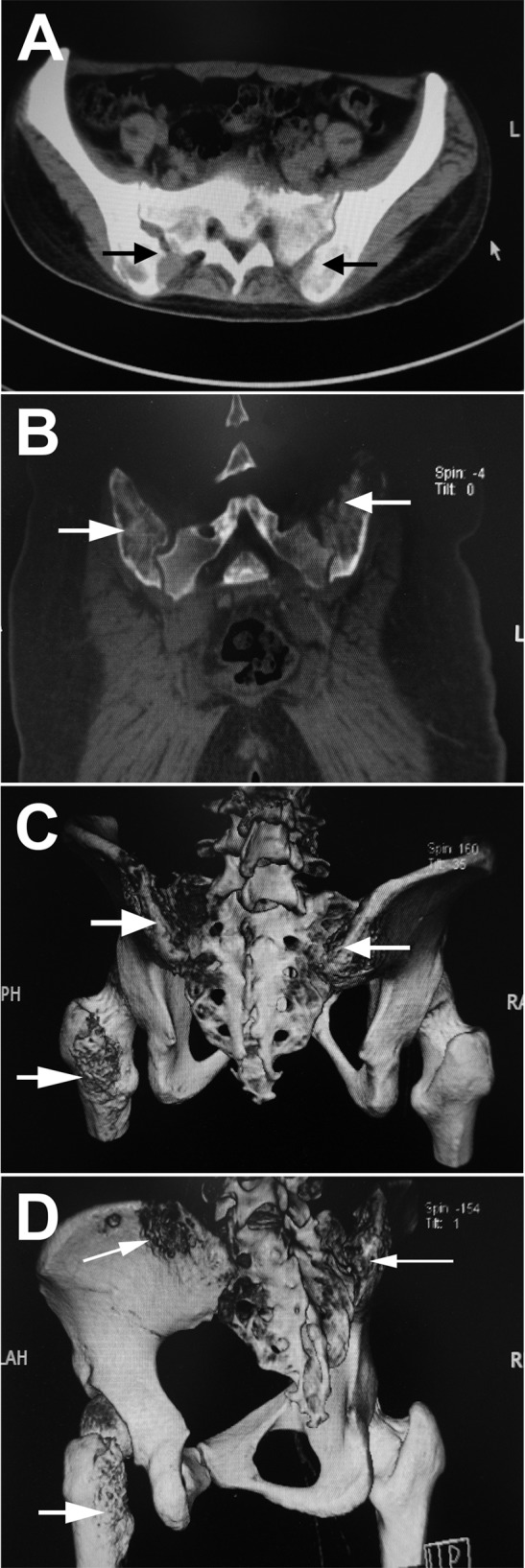
Computed tomography of the abdominal region and pelvis revealing severe osteolysis (arrows) around each sacroiliac joint and the left greater trochanter. However, no extraosseous involvement was visible, such as in the retroperitoneal and hepatic portal lymph nodes. (A) Horizontal position, (B) coronary position and (C and D) three-dimensional reconstructional views.

**Figure 3:**
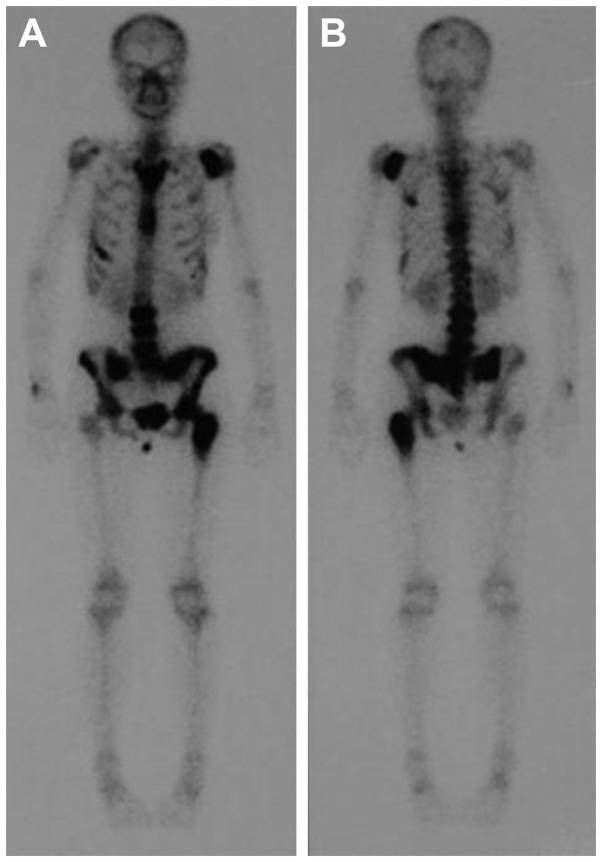
Single-photon emission computed tomography (CT) revealing abnormal bone metabolism of the lumbar, sacroiliac joint, the left greater trochanter and the left shoulder. CT of the lung and mediastinum reveals no abnormalities, except for sclerotin destruction in the left shoulder. (A) Front view and (B) rear view.

**Figure 4:**
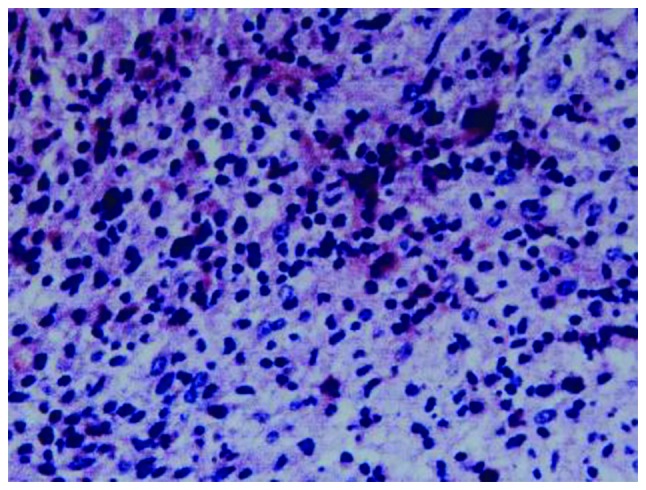
Biopsy sample demonstrating that cells with the typical morphology of Reed-Sternberg (RS) cells, with abundant cytoplasm and prominent eosinophilic nucleoli, were scattered against a background of reactive inflammation. Immunohistochemistry staining of the RS population confirmed the expression of paired box protein-5 and cluster of differentiation (CD)30 and CD15.

Upon presentation, the blood cell count revealed a persistent elevation of white blood cells (>14.0×10^9^ g/l; normal range, 4.0–10.0×10^9^ g/l), and a lowered hemoglobin level (<90 g/l; normal range, 120–150 g/l). The platelet count was also elevated (469.0×10^9^ g/l; normal range, 100–300×10^9^ g/l) and the mean corpuscular volume was <80 fl (normal range, 80–100 fl). The mean corpuscular hemoglobin (MCH) level was <27 pg (normal range, 27–34 pg) and the MCH concentration was <320 g/l (normal range, 320–360 g/l), which suggested microcytic hypochromic anemia. The liver and renal function tests were almost normal; the biochemical evaluation demonstrated signs of inflammation, with levels of 130 mg/l C-reactive protein (CRP) (normal range, 0–8 mg/l) and 0.21 ng/ml procalcitonin (normal range, <0.05 ng/ml), and a 75-mm/h erythrocyte sedimentation rate (ESR) (normal range, 0–20 mm/h). Further serology tests, including the human leukocyte antigen haplotype B27, tuberculosis antibody, light chain protein and extractable nuclear antigens tests, rheumatism immunity and lupus tests, thyroid gland function test and an examination of 12 tumor markers, were all normal. A pelvic X-ray revealed a bone lesion in each of the sacroiliac joints five months after the onset of symptoms (Fig. 1). Computed tomography (CT) identified osteolysis in each sacroiliac joint and the left greater trochanter (Fig. 2). Single-photon emission CT revealed abnormal bone metabolism of the lumbar, sacroiliac joint, the left greater trochanter and the left shoulder (Fig. 3), likely sites of bone metastases. A B-mode ultrasound scan of the cervical and abdominal regions revealed no augmented lymph nodes, and the organs of the abdominal cavity were normal. CT of the lungs and mediastinum, and magnetic resonance imaging of the abdominal region, revealed no enlargement of the lymph nodes and no extraosseous involvement. Scans were combined with a physical examination, which revealed no signs of lymphadenectasis. Positron emission tomography-CT was not recommended due to its high cost and the exposure of the patient to radioactivity. The color ultrasonography images of gynecological features, and the color Doppler ultrasonography of the heart, were normal. Due to the patient’s history of multifocal bone pain and a negative bone marrow biopsy, the clinical profile appeared to be consistent with a diagnosis of chronic recurrent multifocal osteomyelitis (CRMO). Furthermore, the initial biopsy from the left posterior-superior iliac spine also revealed several giant cells against a background of inflammation. Although combination treatment with antibiotics [3.0 g cefperazone-sulbactam, intravenous glucose tolerance test (i.v.g.t.t.), every 8 h; and 1.2 g clindamycin, i.v.g.t.t., every 12 h)] and non-steroidal anti-inflammatory drugs (100 mg flurbiprofen, i.v.g.t.t., every 12 h) for three weeks greatly improved the symptoms, CT revealed that bone destruction was still occurring. A definitive diagnosis was not reached, as the possibility of inflammation, a tumor or even intoxication could not be eliminated. A second biopsy was performed, which retrieved a large amount of tissue for pathological examination. The results of the pathological examination revealed that the tissue contained cells bearing the characteristic morphology of RS cells, with abundant cytoplasm and marked eosinophilic nucleoli, dispersed against a background of reactive inflammation. The immunohistochemistry staining of the RS cell population confirmed the expression of paired box protein-5 and cluster of differentiation (CD)30 and CD15 (Fig. 4). These results led to a diagnosis of primary multifocal osseous lymphoma, rather than CRMO. Finally, in the hematology ward, the patient was prescribed a course of Adriamycin, bleomycin, vinblastine and dacarbazine (ABVD) chemotherapy, and recovered with no pain or fever. The patient was also able to walk normally subsequent to chemotherapy. The biochemical examination revealed that the patient’s CRP, ESR and procalcitonin levels had returned to normal. Furthermore, CT identified no further progression of bone destruction of the lumbar, sacroiliac joint, the left greater trochanter and the left shoulder. The patient returned to the hospital six months later for a follow-up consultation, and reported no pain or fever.

## Discussion

Primary osseous HL is the diagnosis assigned to patients who have osseous HL with no associated extraosseous involvement. If more than one osseous site is involved, a diagnosis of primary multifocal osseous HL is made (2). Primary osseous HL that is limited to the bone is extremely rare, and until 2009, only 16 cases had been identified globally (10). To the best of our knowledge, ~20 cases of primary osseous HL, including 13 patients with primary solitary osseous HL and seven patients with primary multifocal osseous HL without lymphatic manifestations, have been reported (11–17). Li *et al* (18) reported ~30 HL patients with extraosseous involvement. Langley *et al* (2) reported that 33 cases of primary osseous lymphoma at single or multiple sites, in a variety of patient ages, existed in the scientific literature between 1927 and 2008. However, due to a lack of imaging equipment, it cannot be confirmed whether certain patients presented with evidence of lymphadenopathy within the chest or abdomen.

The case reported in the present study was the first case of primary multifocal osseous HL, without associated extraosseous involvement, from China. The extent of osseous involvement included the left shoulder, the inferior segment of the lumbar spine, the two sacroiliac joints and the left greater trochanter. The affected region was wide and confined to the trunk bone beside the mediastinum, however, the mediastinum appeared normal according to a CT image of the thoracic region. The wide range of involvement in unconnected areas had not previously been described in other primary osseous HL cases. The ABVD chemotherapy led to an improvement of the patient’s condition in a short period of time, which suggested that this particular treatment regimen is effective for classical, as well as primary multifocal osseous HL.

According to the Ann Arbor classification system (19), primary osseous HL is classified as stage I, whereas systemic HL with secondary bone involvement is classified as stage IV (18). Therefore, it is important that cases of HL with an unusual presentation are diagnosed correctly by clinicians, as an inaccurate diagnosis may lead to delays in treatment. The histological, radiological and clinical features of HL may mimic those of other medical conditions, including tuberculosis, unusual eosinophilic granuloma, multiple myeloma and infection with human immunodeficiency virus (HIV). There are various types of extra-nodal manifestations of HL besides an osseous presentation. Li *et al* (18) reported the case of a 38-year-old female who presented with pain in the right upper-side of the chest and adjacent soft-tissue swelling for three months. A surgical biopsy, which included morphological and immunological data, was consistent with classical HL, however, the patient had an associated extraosseous soft-tissue mass adjacent to the ribs. Kämmerer *et al* (4) presented the case of a 73-year-old male with a suspicious ulcerating lesion in the left retromolar region of the mandible. Prior anti-inflammatory therapy was unsuccessful and three subsequent biopsies identified inflammation alone. Ultimately, two biopsies from the left retromolar region and the left inner cheek revealed Hodgkin-Steinberg cells that were positive for the expression of CD15 and CD30, a finding which corresponded to a diagnosis of HL. Gandhi *et al* (3) described a case of primary classical HL of the ileum. HL may also occur in the epidural space (20) and in the intracalvarium (21). A case of autoimmune hemolytic anemia and immune thrombocytopenia of HL has also been reported (22). HL not only presents at various extra-nodal sites, but could also be associated with HIV (23). These patients usually have mixed cellularity, or a lymphocyte-depletion subtype, advanced and extra-nodal disease, and systemic symptoms (1). HL may also occur in combination with other diseases, such as multiple myeloma (24), acute leukemia (25), peripheral T-cell lymphoma (26) and splenic marginal zone lymphoma (27). Therefore, HL can occur at various sites adjacent to the skeleton and may be associated with, or occur in combination with, other conditions. Primary osseous HL is extremely rare, and there is a possibility that clinicians may not reach the correct diagnosis.

The present study indicates that, in the case of complex multiple bone disease, imaging may not be sufficient to enable a clear diagnosis. In order to improve the success rate of diagnosis, pathological examination is necessary. During biopsies, doctors should attempt to retrieve as much of the diseased tissue as possible from the multiple lesions, and perform separate pathological examinations in order to improve the success rate of diagnosis. When a pathological diagnosis cannot be confirmed by an initial biopsy, a second biopsy should be performed as soon as possible.
